# The ‘Stolen Generations' of Mothers and Daughters: Child Apprehension and Enhanced HIV Vulnerabilities for Sex Workers of Aboriginal Ancestry

**DOI:** 10.1371/journal.pone.0099664

**Published:** 2014-06-13

**Authors:** Putu Duff, Brittany Bingham, Annick Simo, Delores Jury, Charlotte Reading, Kate Shannon

**Affiliations:** 1 Gender and Sexual Health Initiative, British Columbia Centre for Excellence in HIV/AIDS, Vancouver, British Columbia, Canada; 2 School of Population and Public Health, University of British Columbia, Vancouver, British Columbia, Canada; 3 Faculty of Health Sciences, Simon Fraser University, Vancouver, British Columbia, Canada; 4 Sex Workers United Against Violence (SWUAV), Vancouver, British Columbia, Canada; 5 School of Public Health and Social Policy, University of Victoria, Victoria, British Columbia, Canada; 6 Department of Medicine, University of British Columbia, Vancouver, British Columbia, Canada; Indiana University and Moi University, United States of America

## Abstract

**Objectives:**

The number of children in care of the state continues to grow in BC, Canada with a historical legacy of child apprehension among criminalized and marginalized populations, particularly women of Aboriginal ancestry and sex workers. However, there is a paucity of research investigating child apprehension experiences among marginalized mothers. The objective of the current analysis is to examine the prevalence and correlates of child apprehensions among female sex workers in Vancouver, Canada.

**Methods:**

Analyses were drawn from the AESHA (An Evaluation of Sex Workers Health Access, 2010-present), a prospective cohort of street and off-street SWs, through outreach and semi-annual visits to the research office. Bivariate and multivariate logistic regression were used to examine correlates of child apprehension.

**Results:**

Of a total of 510 SWs, 350 women who had given birth to at least one child were included in the analyses (median age = 37 yrs: IQR: 31–44 yrs). The prevalence of child apprehension among mothers was 38.3%, with 37.4% reporting having been apprehended themselves by child welfare services. In multivariable analysis, servicing clients in outdoor public spaces (versus formal sex work establishments or informal indoor settings) (adjusted odds ratio, (aOR) = 2.73; 95%CI 1.27–5.90), history of injecting drugs (aOR = 2.53; 95%CI 1.42–4.49), Aboriginal ancestry (aOR = 1.66; 95%CI 1.01–2.74) were associated with increased odds of child apprehension.

**Discussion/Conclusions:**

Child apprehension rates are high, particularly among the most marginalized sex workers, including sex workers who use drugs and sex workers of Aboriginal ancestry. Structural reforms to child protection are urgently needed, that support family-based care address the historical legacy of colonization affecting Aboriginal peoples.

## Introduction

The mandate of child protection services is to protect children from neglect and abuse, while supporting the integrity of families [1]. Unfortunately, growing evidence from around the world indicates that current child protection polices are falling short of their goals to protect children and their families For example, a number of studies have reported over-crowding of foster homes, lack of connection with birth parents, neglect and in some cases sexual abuse [2]–[8]. In North America, child protections services have targeted the most marginalized families, including Indigenous peoples (people of Aboriginal ancestry), visible minorities and impoverished families [4], [9], while also falling short of providing families with the support they need to keep children within their care [9], [10]. Sex workers who are parents (i.e., parenting sex workers) experience multiple and intersecting marginalizations, including poverty, homelessness, substance use, lack of social support and violence from clients and intimate partners [11]–[13], that may place them at increased risk for child apprehension. The limited studies that have addressed parenting among sex workers have been qualitative, and focused on broader challenges of parenting rather than child apprehensions specifically. A U.S.-based study among street based sex workers found that just under 10% of sex workers had custody of their children at the time of the interview [14]. This study, along with a number of other qualitative studies, suggests that many sex workers face an ongoing battle to keep their children, with frequent removal of their children by child protection services [14], [15]. For many sex workers, child apprehensions have been found to set in motion a downward spiral into depression, often resulting in escalating drug and alcohol use [16], [17]. Studies among apprehended children of non-sex working low income women suggest that children themselves may also be negatively impacted by separation from their parents, with increased levels of substance use, incarceration, and mental health problems among children who spent time in foster care [5]–[8], [18]. Of concern, a qualitative study from the US suggests cyclical patterns of child apprehension, with many sex workers' separated from their children reporting abandonment or apprehension from their parents in their youth [14].

In Canada, children from low socioeconomic backgrounds are overrepresented in the child welfare system, with apprehension rates being highest among impoverished single mothers, people with mental health, addictions, victims of domestic violence and women of Aboriginal ancestry, the intersection of which contributes to increased vulnerability [10].

In Canada, children of Aboriginal ancestry are currently overrepresented in the child welfare system. While children of Aboriginal ancestry comprise only 8% of the children in British Columbia, they represent 51% of children in care [9]. This overrepresentation may be traced back to a series of colonial processes first implemented in the 1870's [19], such as the separation of children from their parents and communities and placement in residential schools between 1874 and 1986. Residential schools were developed with the aim of assimilating Aboriginal children to the Western society and culture. In residential schools, children were forbidden to speak their ancestral language, and encouraged to be ashamed of their culture and heritage. It is well documented that many children were subjected to various forms of abuse (i.e., physical, sexual and emotional) and neglect by residential school staff [20]. Additionally, in the 1960's, large numbers of children of Aboriginal ancestry were removed from their families and placed for adoption by child welfare agencies [20]–[22]. The legacy of colonialism, as well as multiple and intersecting structural inequities that further marginalized women of Aboriginal ancestry, may continue to drive the overrepresentation of children with Aboriginal ancestry in the child welfare system [23] and contribute to their overrepresentation within the most visible and stigmatized aspects of sex work [24], [25].

Given the dearth of literature surrounding child apprehension among sex workers, including sex workers of Aboriginal ancestry, this study aimed to investigate correlates of child apprehension among street-involved and off-street female sex workers in Vancouver, Canada. In light of the Canadian literature on child apprehensions and ongoing concerns of disproportionate representation of Aboriginal children within child welfare systems, we hypothesize that Aboriginal ancestry will be independently linked to child apprehension, even after adjustment for other risk factors such as injection drug use and homelessness.

## Methods

### Study Design

Known as An Evaluation of Sex Workers Health Access (AESHA), this longitudinal community-based cohort builds on collaborations since 2004. We have previously published on the development and community-based research processes of this project in detail [26]. The project includes a Community Advisory Board with representatives from over 15 community agencies that represent women, Aboriginal and sex work organizations, as well as key stakeholders (health authorities). Research is shared through both academic and non-academic mechanisms (e.g., community, policy venues), and community is invited to provide input on new and emerging research questions, results and interpretation for policies and programs. AESHA initiated recruitment of the prospective cohort of street- and off-street sex workers in Metro Vancouver in 2010, with the aim to document the individual, interpersonal, social, physical and structural environments shaping sexual health and HIV vulnerabilities and health care access and outcomes among sex workers. Eligible participants were aged 14 or older, self-identified as female (transgender inclusive), exchanged sex for money within the past month and provided written informed consent. Time-location sampling was used, which is a probability-based method which recruits participants at times and spaces where they are known to congregate, and uses physical spaces rather than individuals as the primary sampling unit [27]. To identify physical spaces for recruitment, community mapping was conducted by our outreach team that including current/former sex workers. Our outreach team identified and contacted participants at a variety of street and off-street sex work venues: (e.g., street-based sex work “strolls” (e.g., alleys, parks); indoor informal venues (e.g., bars, saunas, hotels); formal sex work establishments (e.g., massage parlours, micro-brothels, or other in-call locations); and online/advertising spaces, such as newspapers). Participants were recruited both during the day and at night at off-street self-advertising spaces (e.g. online, newspapers), indoor sex work establishments (i.e., massage parlours, micro-brothels, in-call locations) and outdoor sex work venues (i.e., streets, alleys, parks).

Following outreach to sex work venues, women were invited to one of the project offices (or a safe, confidential space) to receive more information about the project and complete a detailed informed consent. Following informed consent, women completed a detailed interview-administered questionnaire by trained interviewers (both women with and without previous sex work experience). The baseline questionnaire elicits information related to: socio-demographic characteristics (e.g., age, ethnicity, sexual and gender identity, housing, mobility), sex work patterns (e.g., number of clients, fees, types of services, client characteristics, condom use), sexual health and violence experiences, and physical, social and structural features of the work and living environment (e.g. management policies, policing, security, access to services). Details about criminalization, policing, and ministry/justice system involvement were gathered through questions on history of arrest and incarceration, adverse police encounters without arrest, and previous time spent in foster care. As part of the extensive pre-test counseling questionnaire, questions on sexual and reproductive health and service utilization were asked by the nurse to facilitate support and referral. Remuneration of $40 CAD was provided to participants following each visit to compensate for travel expenses, time and expertise. The remuneration was based on suggested appropriate remuneration of time and expertise, at the suggestion of our community partners and advisory board, including sex work agencies and approved by our research ethics board at the University of British Columbia.

### Ethics

The project holds ethical approval with the Providence Health Care/University of British Columbia Research Ethics Board.

### Child Apprehension

Our dependent variable of interest was history of apprehension by child welfare services, defined as having answered ‘yes’ to the following question: “Have you ever had any children apprehended by Child Welfare Services”?

### Explanatory Variables

The independent variables in this study were chosen based on their known or a priori hypothesized relationship with child apprehension. Socio-demographic characteristics examined included: age (years) as a continuous variable; education (defined as high school graduate versus not), migrant/new immigrant vs. Canadian born, and being of Aboriginal ancestry refers to the Indigenous peoples of Canada: First Nations, Métis and Inuit peoples. It is acknowledged that the Indigenous peoples of Canada represent diverse cultures and languages however, for the purposes of this analysis, the term Aboriginal will be used to refer to these groups collectively as in previous publications [25]. Structural and social factors included ever homeless (defined as having ever spent one night or longer sleeping on the street), workplace (primary place of solicitation and servicing clients) and physical or sexual violence by clients (bad date). Encounters with the justice system included having ever been charged/arrested, spent time in prison or jail, apprehended as a child or spent time in foster care.

### Statistical Analyses

Analyses were restricted to all women who reported giving birth to at least one living child in their lifetime. A complete case analysis approach was used to handle missing data, due to low levels of missingness (<5%). Descriptive analysis for our sample included frequencies, proportions, medians and interquartile ranges (IQR) for continuous data. Bivariable analysis was conducted using Pearson's chi-squared tests for binary categorical variables, and Wilcoxon rank-sum test for continuous variables. To measure strength of association between categorical variables, Odds Ratios (ORs) with 95% Confidence Intervals (CIs) were provided. Fisher's exact test was used when cell sizes were low (<5). Variables with p- values of <0.05 were considered for inclusion in the multivariable model, and Akaike's Information Criteron (AIC) selection was used to arrive at the final model. Variables were considered significant if they maintained p values<0.05 after adjusting for covariates in the multivariable model. The final model was tested for multicollinearity.

## Results

Of 510 street and off-street sex workers at baseline, 350 women reported giving birth to one or more live children and were included in this analyses, Of 350 sex workers, 134 (38.3%) had ever had a child apprehended by Child Welfare Services (CWS). The median age of the entire sample was 38.0 years (Interquartile Range (IQR): 31–44). One third of the entire sample were of Indigenous/Aboriginal ancestry (32%), and 34% were immigrant/new migrant workers (primarily Asian) (**See** **Table 1**).

**Table 1:** Sample characteristics, and bivariate odds ratios for 350 street- and off-street female sex workers who are mothers, stratified by ever having their children apprehended by child welfare services.

		Child apprehended		*p-*value
Characteristic	*Total* *350* *(100%)*	Yes 134 (38.3%)	No 216 (61.7%)	
**Age**		37 (22–53)	39 (19–61)	0.153
**Ethnicity**				
Caucasian	122 (34.9)	50 (37.3)	72 (33.3)	0.450
Visible minority†	228 (65.1)	84 (62.7)	144 (66.6)	
Aboriginal Ancestry	149 (42.6)	79 (59.0)	70 (32.4)	<0.001
Immigrant to Canada	80 (22.9)	5 (3.7)	75 (34.7)	<0.001
High school graduate	145 (41.4)	51 (38.1)	94 (43.5)	<0.001
Homeless*	254 (72.6)	124 (93.2)	130 (60.5)	<0.001
Serviced clients in outdoor settings*	241 (68.9)	121 (90.3)	120 (55.6)	0.001
Client-perpetrated physical or sexual violence*	237 (67.7)	107 (79.5)	130 (60.2)	<0.001
Incarcerated*	228 (65.1)	110 (82.1)	118 (54.6)	<0.001
Apprehended as child*	131 (37.4)	68 (50.8)	63 (29.2)	<0.001
Parents/family spent time in residential school*	98 (28.0)	51 (14.6)	47 (21.8)	0.001
Injection drug use*	201 (57.4)	106 (75.0)	95 (60.0)	<0.001
Received trauma counselling support following child apprehension	37 (10.6)	37 (70.4)	0 (0.0)	

*Ever.

IQR: Interquartile Range.

† Visible minority was defined as being Non-Caucasian, including: Chinese/Taiwanese, South Asian, Pakistani, Bangladeshi, Vietnamese, Korean, Japanese, Filipina, Thai, Sri Lankan, Latin American, Middle Eastern, Black or First Nations.

In bivariate analysis, having ever used injection drugs (odds ratio (OR) = 4.82; 95%Confidence Interval (95%CI) 2.94–7.92), and being of Aboriginal ancestry (OR = 3.00; 95%CI 1.91–4.69) were among the individual level factors that were significant at p<0.05. In our final multivariable model (**Displayed in** **Table 2**), ever serviced clients in outdoor settings (Adjusted odds ratio (AOR) = 2.73; 95%CI 1.27–5.89), ever used injection drugs (AOR = 2.53; 95%CI 1.42–4.50), and Aboriginal ancestry (AOR = 1.66; 95%CI 1.01–2.74), retained statistical significant at p<0.05 after adjusting for potential confounders. Having been apprehended as a child was also marginally significant (at a level of p<0.10) in our final multivariable model (AOR = 1.48; 95%CI 0.90–2.43), and age was not statistically significant (AOR = 1.00 (95% 0.97–1.03)).

**Table 2:** Unadjusted Odds Ratios (ORs) and Adjusted Odds Ratios (AOR) for the independent relationship between individual, social- structural factors and having a child apprehended by child welfare services among 350 parenting female sex workers living in Vancouver.

Characteristic		Odds Ratio (OR)		
	Unadjusted OR (95% CI)	p-value	Adjusted OR (95% CI)	p-value
**Individual-level factors**				
Age	0.98 (0.96–1.01)	0.153	1.00 (0.97–1.03)	0.905
Aboriginal Ancestry	3.03 (1.94–4.73)	<0.001	1.66 (1.00–2.74)	0.049
Immigrant to Canada	0.07 (0.03–0.19)	<0.001	–	–
High school graduate	0.47 (0.31–0.74)	<0.001	–	–
Used injection drugs*	4.82 (2.94–7.92)		2.52 (1.42–4.50)	0.002
**Social- and Structural-level factors**				
Homeless*	9.00 (4.34–18.7)	<0.001	–	
Service outdoors*	7.45 (3.96–14.0)	0.001	2.74 (1.27–5.89)	0.010
Experienced physical or sexual violence by client*	2.62 (1.59–4.33)	<0.001	–	
Arrested/Charged*	1.97 (1.22–3.16)	0.012	–	–
Spent time in jail*	3.81 (2.27–6.38)	<0.001	1.44 (0.77–2.69)	0.249
Apprehended as child*	2.50 (1.60–3.92)	<0.001	1.47 (0.88–2.45)	0.139
Parents/family spent time in residential school	2.21 (1.37–3.55)	<0.001	–	–

*Ever.

## Discussion

These findings reveal high levels of child apprehensions among sex workers, with over a third of women reporting having their children forcibly removed from them. This study is among the first quantitative studies to examine the correlates of child apprehensions among marginalized and stigmatized populations such as sex workers.Our findings lend support to existing qualitative studies that suggest there is a cyclical nature to child apprehensions; sex workers who had been apprehended as a child had 48% increased odds of having their children taken away from them, though this only retained marginal significance when adjusted for other factors in multivariate analysis, suggesting other mechanisms may drive this risk. Elevated odds of child apprehension were also found among sex workers who serviced clients in outdoor/public settings, and sex workers with a history of injection drug use. These findings confirm child welfare reports from British Columbia that demonstrate high levels of child apprehension among marginalized populations [23]. These findings may also reflect child welfare practices that assume, without evidence, that parents involved in sex work are placing their children at increased risk for sexual harm or exploitation, though it is unclear what proportion of apprehensions were based on this evaluation.

Servicing clients in outdoor settings was associated with over a two and a half-fold increased odds of child apprehension compared to women who serviced their clients in indoor venues. While data on parenting among formal indoor sex workers are lacking, these findings agree with a qualitative study that parenting street-based sex workers highly value anonymity with regards to their work [17]. The visibility of outdoor street-based sex workers may also increase their chances of being identified as a sex workers, reported to and targeted by child welfare laws that confuse parental sex work with risk of child sexual abuse and exploitation [28]. Servicing clients in outdoor (e.g., streets, cars, public washrooms, parking lots) or informal indoor settings (e.g., hotels, clients' homes) may also act as proxy for poverty, lack of housing [29], violence and lower social support, all of which increase risk for child apprehension. Child apprehension has been found to exacerbate these vulnerabilities in some cases: loss of governmental child support following apprehension reduces women's income, driving some women further into poverty and sometimes homelessness [10], and can further reduce a woman's chances of regaining custody of her child. This highlights flaws in the current system, and the need to scale up support for sex workers who are mothers. These findings also lend support to the decriminalization of sex work, including the bawdy house provision that prevents women from working in formal, managed indoor settings (e.g., massage parlours and micro-brothels) that would offer enabling environments for sex workers to keep and raise their children. Furthermore, enabling sex workers to work in safer indoor settings will reduce their chances of being identified as a sex worker in the short term, though the larger policy implications of using sex work as grounds for child apprehension remains critical to address. Additionally, removal of the communication provision (prohibiting communicating for the purposes of sex work) will likely improve sex workers' access to parenting services; sex workers often avoid central areas where health and support services exist, to reduce their chances of arrest for soliciting clients [30].

Our findings demonstrate a 66% increased odds of child apprehension among sex workers of Aboriginal ancestry, with 59% of sex workers of Aboriginal ancestry having had a child apprehended, supporting provincial statistics that indicate 51% of children involved in British Columbia's child welfare services are Aboriginal [31]. While previous studies have noted sex workers of Aboriginal are often work in more visible street-based location [25], the strong association with Aboriginal ancestry remained even after adjusting for street-based sex work. This suggests that other mechanisms, such as poverty and perhaps racist practices, may be contributing to the overrepresentation of children of Aboriginal ancestry in care of the state. While further research is needed to elucidate the (direct and/or indirect) links between Aboriginal ancestry and child apprehensions, scholars have speculated this trend is an accumulation of harms resulting from colonization [19], [32]. For example, assimilationist policies, including residential schools and the 60's scoop, tore communities apart, stripping Aboriginal people of their self-, community- and cultural- identities, both at the individual and collective levels [33]. Such policies systematically disempowered people of Aboriginal ancestry, and limited their decisions about their own families and communities [20].

Additionally, residential schools that separated children from their families raised children in an environments lacking healthy parental role models and may have diminished survivors' capacity to parent their own children [20]–[22]. Some authors contend Aboriginal communities continue to experience cultural genocide, evidenced by the fact that more Aboriginal children are under government care today than there were at the height of the residential school era [21]. Moreover, due to a shortage of Aboriginal-specific programming and foster homes, many Aboriginal children are placed in culturally inappropriate homes [9], [20], [21], fostering further cultural disconnect and intergeneration trauma.

Decolonization approaches that emphasize collective healing from intergenerational trauma of colonization, reclaiming of self-, community- and cultural identity, and also promote self-determination may facilitate the healing process. [20], [21]. Supporting self-determination and leadership of people of Aboriginal ancestry is critical to foster the process of decolonization and break the continuous cycle of governmental intrusion into the lives of Aboriginal women. From the perspective of decolonization theory, the ‘over-involvement’ of government represents an attempt to colonize Aboriginal families and further perpetuates colonial oppression [34]. Recently, some control over child welfare services has been handed over to Aboriginal communities, however this differs by jurisdiction [35]. Though this is a step in the right direction, funding and resources to provide family support services remain limited [36].

Funding needs to be allocated to Aboriginal-specific and Aboriginal-led child protection services that focus on the needs of Aboriginal mothers and their children, and embrace traditional parenting practices, culture and knowledge. A special emphasis on restoring community connections, culture, language and pride is also necessary to address the loss of connection resulting from colonization. Greater effort on placing Aboriginal children with extended family, and providing those families with continued support (e.g., financial, parenting supports) necessary to adequately care for the child is needed [9]. Additionally, increasing the number of high quality Aboriginal foster homes will help ensure that children who are removed from their parents are placed in culturally appropriate homes that provide opportunities for children to remain connected with their communities and extended families. This is particularly true for Aboriginal sex workers, who face numerous, intersecting structural inequities, including the legacy of colonialism. While Aboriginal-specific services are required, there remains a need to increase the cultural safety and competence of existing child welfare services that are utilized by Aboriginal families.

The high prevalence of child apprehensions among sex workers who have ever used injection drugs was not unexpected. In Canada, social workers are responsible for assessing the fitness of parents, and use a standardized assessment tool (a check-list of parental characteristics deemed to be risky) to guide their decision. Parental addiction to drugs and alcohol are among the characteristics on this list used to that categorizes mothers as risky parents. A number of qualitative studies suggest that many sex workers are not properly counseled or supported following the removal of their child, with many turning to drugs as a coping mechanism [10]. Moreover, fear of child apprehensions often instills a sense of distrust towards government services [16], [37], further reducing sex workers' already limited access to health and support services. Low-barrier, harm reduction focused programs targeting sex workers that move away from demonizing drug-using women, and instead seek to support the intersecting vulnerabilities of sex workers (e.g., poverty, mental health, substance use, trauma) are urgently required. Parenting programs offer a unique opportunity to reach and treat drug-using sex workers, as many are motivated to quit drugs for their children's wellbeing. Finally, our results show that few women (30%) receive counseling to deal with the trauma of losing their child(ren). There is a need to better support mothers with their loss, as well as continued contact and support from child protection services, particularly for sex workers who aim to regain custody of their children [38].

While child protection services have a responsibility to provide protection and support to children, there is a need to ensure this does not happen at the expense of the community and family integrity. Given that drugs, sex work stigma, and fear of child apprehension have been reported as formidable barriers to health care and parenting services for sex workers [10], there is a need for non-judgmental, women-centred, harm-reduction focused services and supports for sex workers with children. An example of an effective model is Vancouver's Sheway program, which provides low-barrier drug treatment services, health care, and parental support to mothers and their families (until children reach 18 months of age). Aboriginal mothers accessing Sheway services applauded the holistic approach taken by the program, which is aligned with Aboriginal notions of a ‘healing place' [39]. The program has been shown to help women retain/regain custody of their children, increase health care access and improve nutritional status [39]. While these services been beneficial, Aboriginal mothers (many of whom were also sex workers) from the Downtown East-side highlighted a need for enhanced services for children, extended hours and access to parenting support and education programs. Many explained they would benefit from this kind of support, since many did not have a family, or a positive parenting experience from which to draw parenting skills [40]. The provision of 24-hour services by a non-judgmental community-based support worker (outside of the child protections system), has been suggested as a potential solution by sex workers who are mothers [38].

### Limitations

Due to the hidden nature of sex work, attaining a probabilistic sample is challenging. Time-space sampling [27], is a common method to sample hidden populations, and was used to temper this limitation. Social desirability bias resulting in an underreporting of child apprehension may have been an issue given this is a stigmatized and painful issue for many women. However, the community-based participatory practices (e.g., the development of the study and dissemination of findings in partnership with sex work agencies), good rapport of sex workers with the project and interviewers may have reduced this bias. Despite the potential for social desirability bias, which would dilute the association of interest, high numbers of apprehensions, and significant associations with child apprehension remained. Attendance of a family member in residential schools may also be susceptible to under-reporting, due to stigmatization, a culture of silence around the residential school era or unawareness. Given the cross-sectional nature of this study, directionality cannot be confirmed. As mentioned in the discussion, this may be particularly important for variables such as injection drug use, as child apprehension may precede and contribute to the escalation of injection drug use.

In conclusion, we found high levels of child apprehension, concentrated among women of Aboriginal ancestry, and mothers who have ever used injection drugs, suggesting the need for improvements in the child protection system for marginalized and criminalized sex workers. Our results suggest that decriminalization of sex work may improve sex workers' ability to parent and keep their children. Finally, structural reforms to child protection that embrace a decolonization approach may help reduce the cycle of child apprehensions. This includes support of family-based care,with improving parental support for vulnerable mothers (e.g., addictions treatment support, housing).
